# KRAS and TP53 co-mutation predicts benefit of immune checkpoint blockade in lung adenocarcinoma

**DOI:** 10.1038/s41416-024-02746-z

**Published:** 2024-06-12

**Authors:** Jan Budczies, Eva Romanovsky, Martina Kirchner, Olaf Neumann, Miriam Blasi, Johannes Schnorbach, Rajiv Shah, Farastuk Bozorgmehr, Rajkumar Savai, Thorsten Stiewe, Solange Peters, Peter Schirmacher, Michael Thomas, Daniel Kazdal, Petros Christopoulos, Albrecht Stenzinger

**Affiliations:** 1 Institute of Pathology, Heidelberg University Hospital, Heidelberg, Germany; 2 Center for Personalized Medicine (ZPM), Heidelberg, Germany; 3 Translational Lung Research Center Heidelberg (TLRC-H), Member of the German Center for Lung Research (DZL), Heidelberg, Germany; 4 Department of Thoracic Oncology, Thoraxklinik, Heidelberg University Hospital and National Center for Tumor Diseases (NCT), NCT Heidelberg, a partnership between DKFZ and Heidelberg University Hospital, Heidelberg, Germany; 5 Institute for Lung Health (ILH), Justus Liebig University, Giessen, Germany; 6 Max Planck Institute for Heart and Lung Research, Member of the German Center for Lung Research (DZL), Member of the Cardio-Pulmonary Institute (CPI), Bad Nauheim, Germany; 7 Institute of Molecular Oncology, Member of the German Center for Lung Research (DZL), Philipps-University, Marburg, Germany; 8 Department of Oncology, Centre Hospitalier Universitaire Vaudois (CHUV), Lausanne University, Lausanne, Switzerland

**Keywords:** Cancer genomics, Predictive markers, Statistical methods, Non-small-cell lung cancer

## Abstract

**Background:**

Predictive biomarkers in use for immunotherapy in advanced non-small cell lung cancer are of limited sensitivity and specificity. We analysed the potential of activating KRAS and pathogenic TP53 mutations to provide additional predictive information.

**Methods:**

The study cohort included 713 consecutive immunotherapy patients with advanced lung adenocarcinomas, negative for actionable genetic alterations. Additionally, two previously published immunotherapy and two surgical patient cohorts were analyzed. Therapy benefit was stratified by KRAS and TP53 mutations. Molecular characteristics underlying KRASmut/TP53mut tumours were revealed by the analysis of TCGA data.

**Results:**

An interaction between KRAS and TP53 mutations was observed in univariate and multivariate analyses of overall survival (Hazard ratio [HR] = 0.56, *p* = 0.0044 and HR = 0.53, *p* = 0.0021) resulting in a stronger benefit for KRASmut/TP53mut tumours (HR = 0.71, CI 0.55–0.92). This observation was confirmed in immunotherapy cohorts but not observed in surgical cohorts. Tumour mutational burden, proliferation, and PD-L1 mRNA were significantly higher in TP53-mutated tumours, regardless of KRAS status. Genome-wide expression analysis revealed 64 genes, including CX3CL1 (fractalkine), as specific transcriptomic characteristic of KRASmut/TP53mut tumours.

**Conclusions:**

KRAS/TP53 co-mutation predicts ICI benefit in univariate and multivariate survival analyses and is associated with unique molecular tumour features. Mutation testing of the two genes can be easily implemented using small NGS panels.

## Background

Inhibitors of programmed death 1 (PD-1) and programmed death ligand 1 (PD-L1) have revolutionised the treatment of non-small cell lung cancer (NSCLC). In advanced tumours, immune checkpoint inhibition (ICI) prolongs survival in both first-line and second-line settings [1] and represents the current standard-of-care at diagnosis for all patients with advanced NSCLC without actionable genetic alterations [2].

However, despite the potential for durable response, long-term disease control is achieved in only a minority of patients, which has stimulated extensive efforts to identify biomarkers of response and resistance to immunotherapy [3]. Currently, PD-L1 protein expression is the only approved biomarker for ICI in NSCLC in Europe, while tumour mutational burden (TMB) and microsatellite instability (MSI) are additionally approved by the FDA in an entity-agnostic manner. While PD-L1 expression ( ≥ 1% or ≥50%) is mandatory for ICI-monotherapy, combination treatment of ICI with platinum doublets is possible regardless of PD-L1 expression [4]. There is an urgent need for additional predictive biomarkers in NSCLC, as both PD-L1 and TMB are imperfect biomarkers with limited sensitivity and specificity for predicting ICI [5], which still suffer from biological and technical issues impacting standardisation and reproducibility [6–8].

Mutation testing is mandatory in advanced lung adenocarcinoma because patients with actionable genetic alterations such as EGFR mutations and ALK/RET/ROS1 fusions should receive targeted therapy rather than ICI-comprising regimens in the first-line [2]. Several studies have investigated the role of oncogenic mutations in ICI response, but the influence of co-mutations in tumour suppressor genes has not been adequately studied [9]. TP53 and KRAS are the most frequently mutated genes in lung adenocarcinoma populations of Western countries being altered in about half and one-third of patients, respectively [10]. In a recent study of KRAS-mutated lung adenocarcinoma, objective response rates to ICI differed among tumours with STK11/LKB1 co-mutation, tumours with TP53 co-mutation, and tumours without such co-mutations [11]. Frost et al. identified KRAS/TP53 co-mutation as a biomarker for long-term response to pembrolizumab monotherapy in lung adenocarcinoma with high ( ≥ 50%) PD-L1 expression [12, 13].

To investigate the impact of the KRAS and TP53 mutations on the benefit of ICI in an independent and broader patient population, we analysed a large cohort of patients with lung adenocarcinoma from the Thoraxklinik Heidelberg (HD-ICI) and two external cohorts including patients treated with ICI monotherapy and combination therapy of ICI with platinum-based chemotherapy. We detected longer survival for KRASmut/TP53mut tumours compared with tumours with other co-mutation configurations in univariate and multivariate analyses, in most of the analysed subgroups, and consistently in the three analysed cohorts. To gain insight into the molecular characteristics of KRASmut/TP53mut tumours and potential mechanisms contributing to the increased benefit from ICI, we analysed molecular data from The Cancer Genome Atlas (TCGA).

## Material and methods

### Heidelberg cohort

We conducted a retrospective cohort study without randomisation. We analysed a consecutive series of 713 patients with EGFR and ALK/RET/ROS1 negative advanced lung adenocarcinoma treated with ICI at the Thoraxklinik Heidelberg between 2017 and 2023 (HD-ICI, Suppl. 1). The study was performed in accordance with the Declaration of Helsinki and has been approved by the ethics committee of the Heidelberg Faculty of Medicine (vote S-638-2016). Informed consent was obtained from all study participants. Mutation analysis was performed at the Institute of Pathology Heidelberg combining multiplexed PCR and semiconductor sequencing (Ion Torrent S5; Thermo Fisher Scientific, Inc., Waltham, MA, USA) as described before [14]. In brief, targeted DNA sequencing was performed using the custom NGS panels including EGFR, KRAS, and TP53. Targeted RNA sequencing for the detection of gene fusions was performed using Archer (Archer^®^DX, Inc., Boulder CO, USA) as described before [14]. Clinical data including smoking history, PD-L1 protein expression, treatment regimens, progression-free survival (PFS), and overall survival (OS) were obtained from the clinical database of the Thoraxklinik Heidelberg. Patients who progressed or died within six weeks after initiation of ICI were excluded from the cohort because of a very short exposure to immunotherapy and a high probability of death from causes other than disease progression.

### External cohorts

We additionally analysed four external lung adenocarcinoma cohorts with mutation and survival data publicly available (Suppl. 2). Two validation cohorts SU2C-ICI [15] and MSK-ICI [16] of stage IV lung adenocarcinoma patients that received ICI were analysed to replicate the analysis of the study cohort. To separate predictive from prognostic impact, we additionally analysed two reference cohorts TCGA-LUAD [10] and MSK-LUAD [17] of surgically treated stage I-IV lung adenocarcinoma patients that did not receive ICI. Tumours with activating EGFR mutations or activating ALK/RET/ROS1 fusions were excluded from the analyses (except for SU2C, for which no gene fusion information was available). For survival analyses in the external cohorts, patients who progressed or died within six weeks after initiation of treatment were excluded. Suppl. 3 gives an overview of the excluded and included patients in the five cohorts studied.

### Mutation classification

KRAS mutations were classified into activating variants, likely activating variants, variants of unknown significance (VUS), and neutral variants. Tumours were classified according to activating or likely activating mutations (KRASmut tumours) and the remaining tumours (KRASwt tumours). TP53 mutations were classified into gain-of-function (GOF), loss-of-function (LOF), VUS, and neutral variants as described before [18]. Tumours were classified according to GOF or LOF mutations (TP53mut tumours), and the remaining tumours (TP53wt tumours). Lollipop plots of the detected KRAS and TP53 variants were generated using MutationMapper within the cBioPortal platform [19].

### Analysis of clinical-pathological characteristics and survival

The analysed patient and tumour characteristics included age (continuous), sex (male and female), smoking status (smoker and non-smoker), tumour stage, PD-L1 expression, type of therapy and therapy line ICI. Patients’ age varied from 30 to 90 years (median 65 and sd 8.9). The association of mutation status with clinical-pathological characteristics was tested for statistical significance using Fisher’s exact test. The endpoints of progression-free survival (PFS) and overall survival (OS) were examined in univariate and multivariate survival analyses. Hazard ratios (HR) were reported with 95% confidence intervals (CI). The significance of the univariate survival analyses was assessed using the log-rank test. The significances of the parameters in the multivariate survival analyses were assessed using Wald’s test. Statistical analysis and graphics generation were performed using the statistical programming language R and the R package survival [20]. Everywhere, *p*-values smaller than 0.05 were considered significant.

### Molecular analyses

Mutation and gene expression data of TCGA-LUAD were obtained from TCGA pan-cancer homepage of the National Cancer Institute [21]. Tumour mutational burden (TMB) was calculated as the number of missense mutations and bridged to a value per Mb, where 199 mutations correspond to 10 mutations/Mb, as previously described [22]. The abundance of 14 immune cell populations in the tumour microenvironment was estimated from gene expression data as described by Danaher et al. [23]. The association of TMB, TOP2A expression, and the 14 immune cell population and mutation status was assessed using the Kruskal-Wallis test and further analysed post hoc using the two-sided Wilcoxon test.

Differential expression between KRASmut/TP53mut tumours and the three other mutation configurations was analysed using the two-sided Wilcoxon test. Next, *p*-values were corrected for multiple hypothesis testing using the Benjamini–Hochberg method and lists of differentially expressed genes were compiled controlling the false discovery rate (FDR) at 10%. Intersections between the sets of differentially expressed genes were tested for significance using Fisher’s exact test.

Sets of over- and underexpressed genes were analysed for enrichment and depletion of the 50 categories and the GSEA cancer hallmarks catalogue [24]. Significance testing was performed using Fisher’s exact test. Next, *p*-values of the 4×2×50 analyses were corrected using the Benjamini–Hochberg method and the significantly enriched or depleted categories were collected controlling the FDR at 10%. The fold change in enrichment was calculated as the quotient of the proportion of genes annotated in a category of the analysed gene set and the proportion of genes annotated in the category of the genome. Categories with a fold change of enrichment of more than two (or less than half) were considered as strongly enriched (or strongly depleted).

## Results

The study cohort included 713 patients with advanced lung adenocarcinoma from the Thoraxklinik Heidelberg (HD-ICI) treated with ICI (Suppl. 1). To separate predictive from prognostic impact, we additionally analysed a cohort of 417 patients with stage I-IV adenocarcinomas from The Cancer Genome Atlas (TCGA-LUAD) as a reference cohort of patients who did not receive ICI. Tumours with actionable genetic alterations were excluded from both cohorts. Based on tumour genetics, patients were classified into KRASmut/TP53mut, KRASmut/TP53wt, KRASwt/TP53mut, and KRASwt/TP53wt groups. The prevalence of these mutation configurations was 17%, 33%, 23%, and 27% in the HD-ICI cohort and 12%, 22%, 38%, and 28% in the TCGA-LUAD cohort.

### Association of KRAS/TP53 mutation status with clinicopathological characteristics

We analysed the association between KRAS/TP53 status and clinicopathological patient characteristics (Table 1, Suppl. 4). In HD-ICI, tumour genetics correlated significantly with the sex and a similar but non-significant trend was observed in TCGA-LUAD: A higher proportion of female patients (22 and 15%) had KRASmut/TP53mut tumours compared with male patients (13 and 9%). A strong and highly significant association between KRAS/TP53 and smoking status was found in both HD-ICI and TCGA-LUAD: Significantly higher proportions of smokers (19 and 13%) had KRASmut/TP53mut tumours compared with non-smokers (8 and 5%), whereas slightly higher proportions of smokers had KRASmut/TP53wt and KRASwt/TP53mut compared with non-smokers. By contrast, the proportion of smokers (23 and 25%) with KRASwt/TP53wt tumours was significantly lower than that of non-smokers (49 and 56%). In HD-ICI, a significant association was found between PD-L1 protein expression and KRAS/TP53 status, whereas PD-L1 protein expression data were not available for TCGA-LUAD. The percentage of PD-L1 negative tumours in HD-ICI was low for KRASmut/TP53mut tumours (10%), intermediate for KRASwt/TP53mut tumours (25%), and high for KRASmut/TP53wt and KRASwt/TP53wt tumours (both 33%).

**Table 1:** Association of KRAS and TP53 co-mutation status with patients and tumour characteristics in the Heidelberg cohort of 713 stage IV lung adenocarcinoma (HD-ICI).

Variable	Value	KRASwt/TP53wt	KRASwt/TP53mut	KRASmut/TP53wt	KRASmut/TP53mut	*p*
Prevalence		191 (26.8%)	162 (22.7%)	237 (33.2%)	123 (17.3%)	
Age	Mean ± sd	66.2 ± 9.6	65 ± 8.9	64.3 ± 9	64.7 ± 7.7	0.072
Sex						0.0039
	Male	107 (27%)	105 (26.5%)	131 (33.1%)	53 (13.4%)	
	Female	84 (26.5%)	57 (18%)	106 (33.4%)	70 (22.1%)	
Smoking						4.9e-07
	Smoker	137 (22.7%)	141 (23.4%)	211 (35%)	114 (18.9%)	
	Never/light	54 (49.1%)	21 (19.1%)	26 (23.6%)	9 (8.2%)	
PD-L1						0.0035
	≥ 50%	38 (18.4%)	50 (24.3%)	68 (33%)	50 (24.3%)	
	≥ 1–49%	79 (28.3%)	59 (21.1%)	94 (33.7%)	47 (16.8%)	
	negative	66 (32.5%)	50 (24.6%)	66 (32.5%)	21 (10.3%)	

Tumours harbouring EGFR driver mutations or ALK/RET/ROS1 driver fusions were excluded from the cohort.

### Univariate and multivariate survival analysis

**Fig. 1:**
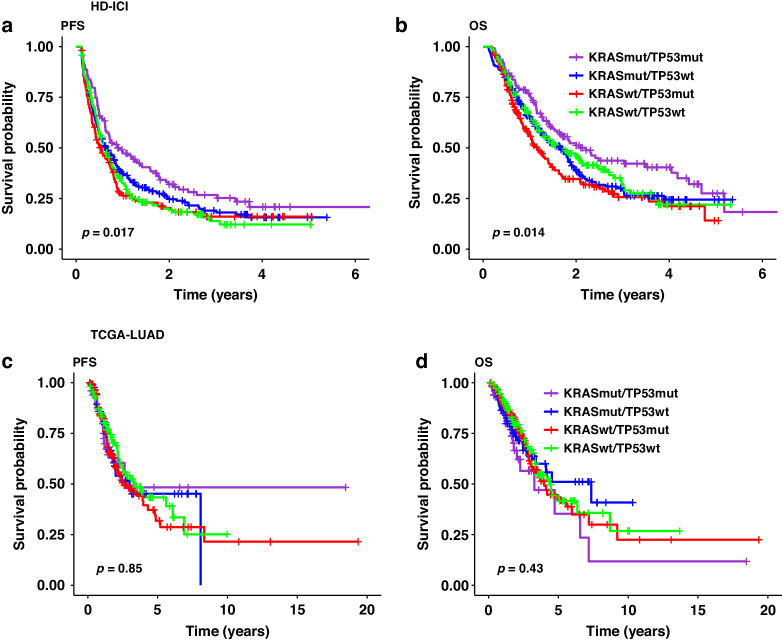
KRAS and TP53 co-mutation status as predictive marker for benefit from ICI in lung adenocarcinoma. **a**,**b** Heidelberg cohort (HD-ICI): prognostic impact of co-mutations status in patients treated with ICI. **c**,**d** TCGA cohort (TCGA-LUAD): absence of the prognostic impact of co-mutations status in patients that underwent surgery.

In patients treated with ICI (HD-ICI cohort), PFS and OS differed between patient groups defined by mutation status (*p* = 0.017 and *p* = 0.014, respectively) and the best clinical outcome was observed in patients with KRAS and TP53 co-mutated tumours (Fig. 1a, b). By contrast, PFS and OS did not differ according to KRAS/TP53 status when no ICI was administered (TCGA-LUAD cohort, Fig. 1c, d). Post hoc analysis in HD-ICI was performed in two different ways: The first model included KRAS and TP53 as two independent variables and an interaction term. The second model included a single four-level categorical variable with KRASmut/TP53mut as the reference category. For both types of modelling, we performed both univariate and multivariate analyses that included age, sex, smoking status, PD-L1 status, type of therapy (ICI as monotherapy or in combination with chemotherapy), and first-line/later-line ICI.

Using the first approach, we found a strong influence of the interaction between the two concomitant mutations on OS in univariate and multivariate analysis (Table 2; HR = 0.56 CI 0.38–0.84, *p* = 0.0044 and HR = 0.53 CI 0.35–0.79, *p* = 0.0021). We also found a limited negative impact of TP53 mutations on OS (HR = 1.29 CI 0.98–1.68, *p* = 0.066 and HR = 1.48 CI 1.11–1.96, *p* = 0.0067), while KRAS mutations had no impact on OS. Analysis of PFS showed results in the same direction (Suppl. 5). Of the other variables analysed, only age and PD-L1 status, but not smoking status and type or line of therapy, had a significant impact on OS (in both univariate and multivariate analyses).

**Table 2:** Influence of KRAS and TP53 co-mutation status on OS in patients treated with ICI.

Variable	Contrast	HR OS univariate	p OS univariate	HR OS multivariate	p OS multivariate
**Tumour genetics**					
	KRASmut	1.08 (0.84–1.38)	0.57	1.21 (0.93–1.57)	0.16
	TP53mut	1.29 (0.98–1.68)	0.066	1.48 (1.11–1.96)	0.0067
	Interaction	0.56 (0.38–0.84)	0.0044	0.53 (0.35–0.79)	0.0021
**Age**					
	Per 10 years	1.16 (1.04–1.29)	0.0073	1.17 (1.05–1.31)	0.0063
**Sex**					
	Female vs. male	0.86 (0.71–1.04)	0.13	1.13 (0.92–1.38)	0.24
**Smoking**					
	Smoker vs. never/light	0.83 (0.64–1.06)	0.14	0.89 (0.67–1.18)	0.42
**PD-L1**					
	≥ 50% vs. negative	0.61 (0.47–0.79)	0.00014	0.58 (0.44–0.78)	0.00024
	≥ 1–49% vs. negative	0.82 (0.65–1.03)	0.091	0.8 (0.63–1.02)	0.068
**Comb. with CHT**					
	ICI + CHT vs. ICI	1 (0.83–1.21)	0.98	1 (0.76–1.31)	0.98
**Therapy line ICI**					
	1st vs. ≥ 2nd line	0.85 (0.7–1.03)	0.091	0.81 (0.63–1.05)	0.11

Univariate and multivariate analyses in HD-ICI.

Using the second approach, we observed numerically worse clinical outcomes for all three other KRAS/TP53 configurations compared with the reference category of KRASmut/TP53mut tumours for both PFS and OS and in both univariate and multivariate analyses (Suppl. 6 and 7). KRASwt/TP53mut tumours showed significantly worse outcomes in each of the four analyses, whereas significance was reached only in the univariate analyses for the other KRAS/TP53 configurations. Overall, the survival analyses support a role for KRAS and TP53 co-mutation status as predictive marker of ICI benefit, with patients with KRAS/TP53 double-mutated tumours archiving the best outcome.

### Subgroup analysis and validation in external cohorts

**Fig. 2:**
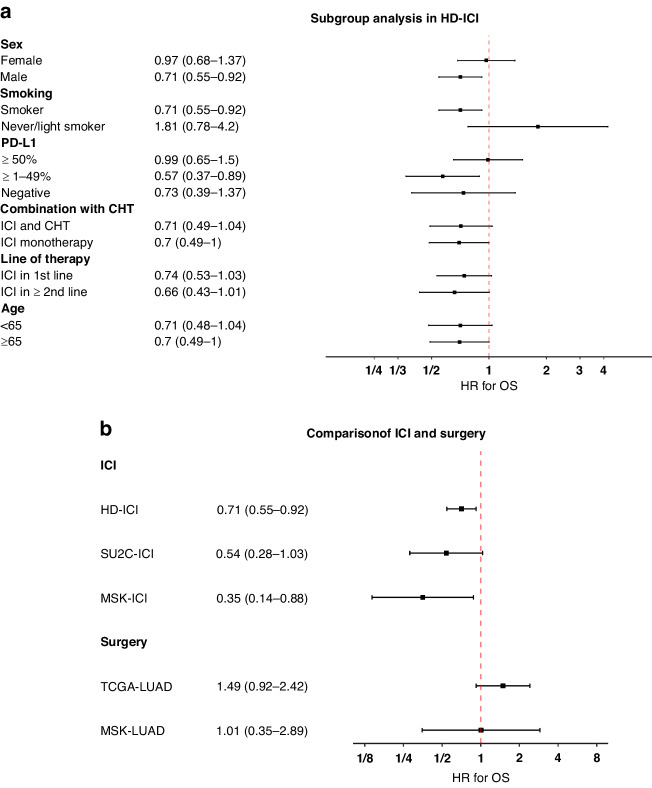
In-depth analysis of the prognostic and predictive impact of dual KRAS and TP53 mutation. **a** Subgroups analysis of HD-ICI confirming longer OS after ICI in most of the analysed subgroups. **b** Longer OS in the study cohort (HD-ICI) and two external cohorts (SU2C-ICI and MSK-ICI) of patients treated with ICI. By contrast, no prolongation of survival was observed in two external cohorts (TCGA-LUAD and MSK-LUAD) of conventionally treated patients.

Subgroup analysis in HD-ICI revealed numerically better survival of patients with double-mutated tumours in almost all subgroups except for non-smokers and of patients with PD-L1 expression of ≥50% (Fig. 2a [OS], Suppl. 8A [PFS]). To validate predictive relevance, we additionally analysed two external cohorts of patients treated with ICI and two cohorts of surgically treated patients (Fig. 2b [OS], Suppl. 8B [PFS]). The increased benefit of ICI for patients with double-mutated tumours observed in the study cohort was confirmed in the two external validation cohorts, HD-ICI: HR = 0.71 (0.55–0.92), SU2C-ICI: HR = 0.54 (0.28–1.03), and MSK-ICI: HR = 0.35 (0.14–0.88) in the analysis of OS and similar results were observed in the analysis of PFS. To separate predictive from prognostic impact, we additionally analysed two external cohorts (TCGA-LUAD and MSK-LUAD) of surgically treated lung adenocarcinoma patients who did not receive ICI. In these cohorts, dual KRAS/TP53 mutation was not associated with better PFS or OS.

### Analysis of specific KRAS and TP53 mutations

**Fig. 3:**
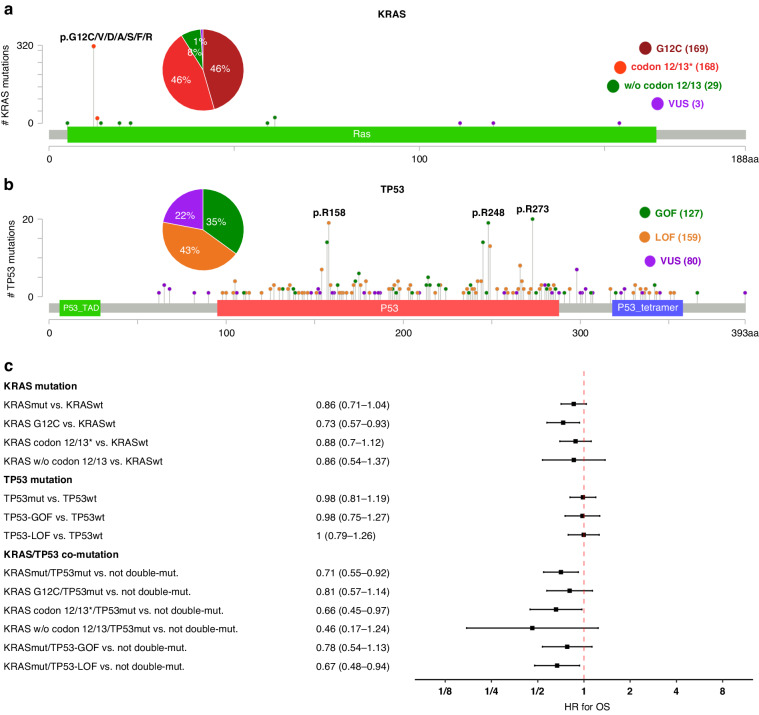
Analysis of KRAS and TP53 mutation types in HD-ICI. **a** Distribution of the mutations in KRAS. **b** Distribution of the mutations in TP53. **c** Univariate analysis of OS comparing tumours having specific KRAS mutations with KRASwt tumours, of the tumour having specific TP53 mutations with TP53wt tumours, and of tumours having specific types of KRAS/TP53 co-mutation with the basket of not double-mutated tumours. *without G12C mutation

Lollipop plots show the mutations detected in KRAS and TP53 in the Heidelberg cohort (Fig. 3A, B). KRAS mutations were classified as G12C mutations (*n* = 169), other activating mutations in codons 12 and 13 (*n* = 168), other activating mutations outside codons 12 and 13 (*n* = 29), and VUS (*n* = 3). TP53 mutations were classified as GOF mutations (*n* = 127), LOF mutations (*n* = 159), and VUS (*n* = 80). Very few KRAS mutations and the minority of TP53 mutations were classified as VUS (KRAS: 1%, TP53: 22%).

We analysed the impact of specific KRAS and TP53 mutations on survival in the Heidelberg cohort (Fig. 3c). All types of activating KRAS mutations were associated with numerically better OS. The strongest association was found for G12C mutations (HR = 0.73, CI 0.57–0.93). By contrast, altered OS was not observed for either TP53 GOF mutations or TP53 LOF mutations compared with TP53wt tumours. Furthermore, patients with all types of KRAS/TP53 double mutations exhibited numerically longer OS compared to patients not harbouring the dual mutation configuration without any apparent differences according to the subtype of KRAS or TP53 mutation. Similar results were detected for PFS (Suppl. 9). In summary, there was no evidence of survival advantages or disadvantages associated with specific types of KRAS or TP53 mutations.

### Molecular characteristics of KRASmut/TP53mut tumours

**Fig. 4:**
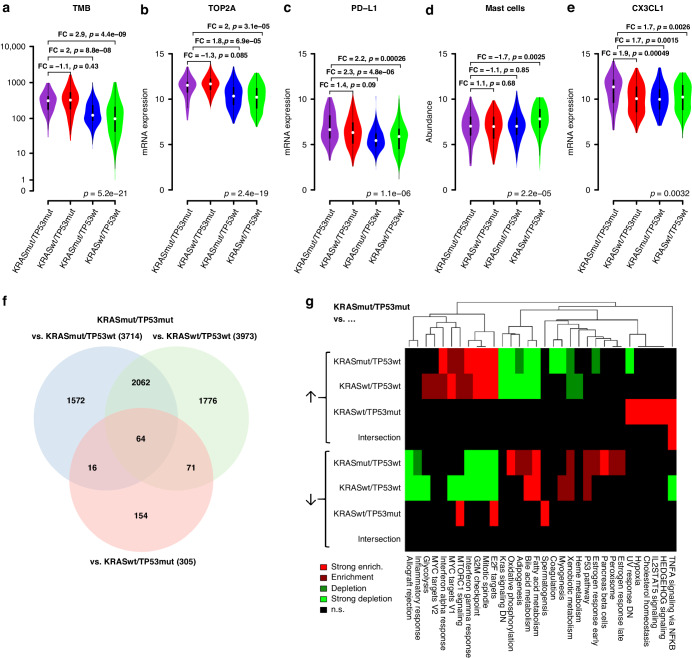
Molecular tumour features associated with KRAS and TP53 co-mutation status (TCGA-LUAD cohort). **a** Association of TMB with co-mutations status. **b–e** Association of TOP2A mRNA, PD-L1 mRNA, mast cells, and CX3CL1 mRNA with co-mutation status. **f** Numbers of differentially expressed genes between KRASmut/TP53mut tumours and KRASmut/TP53wt tumours (blue), KRASwt/TP53wt tumours (green), and KRASwt/TP53mut tumours (red). **g** Heatmap display of the significantly enriched and depleted categories of the GSEA cancer hallmark catalogue in the sets of differentially expressed genes. ↑ = overexpressed genes, ↓ = underexpressed genes

We sought to uncover biological features of KRAS/TP53 double-mutated tumours that contribute to the improved benefit of this tumour type from immunotherapy. To this end, we analysed TMB, TOP2A as a proliferation marker, PD-L1, 14 immune cell populations, and genome-wide expression patterns in the TCGA-LUAD cohort (Fig. 4, Suppl. 10). TMB, TOP2A mRNA, and PD-L1 mRNA were approximately twice as high in TP53mut tumours compared with TP53wt tumours regardless of KRAS status (Fig. 4A-C). Thus, higher TMB, higher proliferation, and higher PD-L1 levels could contribute to the increased benefit of ICI for TP53mut tumours, but do not explain the increased benefit for KRASmut/TP53mut tumours compared with KRASwt/TP53mut tumours.

Five of 14 immune cell populations were significantly associated with co-mutation status (Fig. 4D, Suppl. 10): mast cells, NK cells, exhausted CD8 cells, CD8 cells, and cytotoxic cells (FDR = 10%). Among these, mast cells and NK cells were differentially represented in KRASmut/TP53mut tumours compared with at least one of the other groups. Mast cells were less abundant in KRASmut/TP53mut tumours compared with KRASwt/TP53wt tumours (fold change = −1.71, *p* = 0.0025), but not compared with KRASmut/TP53wt and KRASwt/TP53mut tumours. NK cells were less abundant in KRASmut/TP53mut tumours compared with KRASwt/TP53mut tumours (fold change = −1.25, *p* = 0.03), but not compared with KRASmut/TP53wt and KRASwt/TP53wt tumours. In summary, none of the individual markers examined separated KRASmut/TP53mut tumours from all other KRAS/TP53 mutation configurations.

### Genome-wide expression analysis of KRASmut/TP53mut tumours

Differential gene expression analysis of KRASmut/TP53mut tumours compared with KRASmut/TP53wt and KRASwt/TP53wt tumours revealed a large number of 3714 and 3973 differentially expressed genes, respectively (FDR = 10%, Fig. 4F). By contrast, when KRASmut/TP53mut tumours were compared with KRASwt/TP53mut tumours, a lower number of 305 differentially expressed genes was detected. The pairwise overlaps between the three sets of differentially expressed genes were all highly significant (all *p* < 2.2e-16).

To gain functional insight, the three gene sets and their intersection were analysed for the enrichment or depletion of 50 cancer hallmark gene sets, separately for the over- and underexpressed genes (Fig. 4G). Thirty-two of the 50 hallmark gene sets were significantly enriched or depleted in at least one of the analyses (FDR = 10%). Many enriched and depleted categories were identical in the comparisons of KRASmut/TP53mut with KRASmut/TP53wt tumours and with KRASwt/TP53wt tumours, whereas completely different categories were found in the comparison of KRASmut/TP53mut with KRASwt/TP53mut tumours. In both of the comparisons of KRASmut/TP53mut with TP53wt tumours, the p53 pathway was enriched in the underexpressed genes, whereas three categories related to tumour cell proliferation (G2M checkpoint, E2F targets, and mitotic spindle) were highly enriched in the overexpressed genes. These observations are consistent with LOF of TP53 and the consequent loss of control over the cell cycle in the TP53mut tumours. Six functional categories were enriched in the overexpressed genes between KRASmut/TP53mut and KRASwt/TP53mut tumours, but these pathways were not enriched in any other comparison.

Next, we focused on the intersection of the three gene sets that included 64 genes, most of which were either overexpressed (*n* = 22, 34%) or underexpressed (*n* = 34, 53%) consistently across the three comparisons (Suppl. 11). Out of these, nine genes were annotated to the category “immune system process” (GO:0002376), namely CX3CL1, OAS2, DDX58, and SP110 that were overexpressed as well as ADD2, CTFS, FBXO9, LMO4, and SLC5A5 that were underexpressed across the three comparisons. LMO4, a potential oncogene that has been implicated in prognosis, cell migration, and invasion in NSCLC [25] was among the underexpressed genes, while CX3CL1 (fractalkine) that exists in two forms, either membrane-anchored or as secreted chemokine [26] was among the overexpressed genes (Fig. 4e). Fractalkine can potently attract T cells, NK cells, and monocytes and also adhere to the attracted cells [27]. Thus, overexpression of CX3CL1 may contribute to mediating the increased benefit from ICI in patients with dual KRAS/TP53 mutation.

## Discussion

Current biomarkers for NSCLC patients treated with ICI have limited predictive power and suffer from practical issues related to standardisation and reproducibility. While classic actionable alterations serve as negative biomarkers in daily clinical practice, the only widely used positive predictor is PD-L1 protein expression which at a threshold of of ≥1% and ≥50% is indicative of therapeutic regimes without chemotherapy such as pembrolizumab and atezolizumab monotherapy [4]. In the vast majority of patients receiving various chemo-IO combination therapies, any level of PD-L1 expression is sufficient for initiation of treatment, but the response to therapy varies widely from patient to patient. In this retrospective study, we contribute to overcoming these significant limitations by analysing a large real-world dataset of more than 700 patients with stage IV lung adenocarcinoma who underwent ICI therapy at the Thoraxklinik Heidelberg. The main clinical finding of an increased benefit from ICI for patients with KRASmut/TP53mut tumours was validated in the publicly available datasets SU2C-ICI and MSK-ICI. We also analysed the datasets TCGA-LUAD and MSK-LUAD comprising resected specimens of earlier stages of the disease as a baseline of patients treated without early involvement of ICI.

Consistent with previous reports, we observed an enrichment of KRASmut/TP53mut adenocarcinomas in smokers. Analysis of PD-L1 protein expression revealed that a greater proportion of tumours with PD-L1 expression on ≥50% of tumuor cells showed double-mutated KRAS/TP53 compared to tumours with low and without PD-L1 expression (25% compared to 17 and 10%). Patients with KRASmut/TP53mut tumours had significantly longer OS compared to the other KRAS/TP53 mutation configurations, both in univariate and multivariate analyses. This observation held true for all subgroup analyses except for the PD-L1 ≥ 50% and non-smoking groups. Thus, while expression of PD-L1 expression in KRAS/TP53 double-mutated tumours could contribute to a better prognosis in the univariate analysis, multivariate and subgroup analyses highlight its role as an independent prognostic marker. Better survival was observed for all types of KRAS mutations and for TP53 GOF and LOF mutations. The increased benefit of patients with KRASmut/TP53mut tumours was confirmed in the SU2C-IC and MSK-ICI cohorts, suggesting the validity of the observation across different patient populations.

The predictive value of KRAS and TP53 mutations in lung adenocarcinoma has been investigated in exploratory analyses of data from clinical studies evaluating immunotherapy in NSCLC before, specifically in Checkmate 9LA, Checkmate 227 Part 1, Keynote 042, Keynote 189, and Poseidon [28–32]. First, all of these analyses were compatible with a universal benefit from immunotherapy regardless of KRAS and TP53 mutation status. Second, trends for increased benefit from immunotherapy in patients with KRAS mutations were observed in some of the studies including Keynote 042 and Poseidon - in Poseidon specifically for the patients receiving combined blockage of CTLA4 and the PD-1/PD-L1 axis. However, the impact of KRAS and TP53 mutations remains inconclusive based on these analyses, due to small sample sizes in the mutation subgroups and due to discordances between different studies. It should be noted that the cited clinical studies were not powered for stratification in mutation subgroups. Third, subgroups defined by co-mutation of two or more genes were not analysed in clinical trials evaluating immunotherapies so far. The current study evaluating a large real-world population of lung adenocarcinoma patients confirms and generalises the predictive impact of KRAS/TP53 co-mutation than was reported in two earlier studies in a more specific patient population especially for KRAS G12C mutations [12, 13].

TCGA data were analysed to elucidate the molecular basis of the double-mutated tumours and possible mechanisms contributing to the survival advantage. KRASmut/TP53mut tumours exhibited high TMB, high PD-L1, and high proliferation. But these properties were not specific for KRAS/TP53 double-mutated tumours as they also were observed in KRASwt/TP53mut tumours. The immune TME composition did not exhibit features that simultaneously distinguished double mutation from all other mutation configurations. Genome-wide expression analysis revealed a high number of differentially expressed genes between double-mutated tumours and TP53wt tumours, many of which were attributed to TP53 signalling and tumour cell proliferation, in line with earlier analyses of TP53-mutated tumours [18]. A smaller number of differentially expressed genes was detected between KRASmut/TP53mut and KRASwt/TP53mut tumours, resulting in an intersection of 64 genes that distinguished the double-mutant from all other KRAS/TP53 mutation configurations. This intersection included fractalkine (CX3CL1) which was overexpressed in KRASmut/TP53 tumours compared with all other KRAS/TP53 configurations. Fractalkine encodes a protein that exists in two forms either anchored in the cell membrane or as a secreted chemokine and binds exclusively to CX3CR1, unlike most other chemokines that can bind to multiple receptors [33, 34]. In an analysis of seven expression datasets, CX3CL1 expression was a strong and reproducible positive prognostic marker in lung adenocarcinoma [35]. Furthermore, fractalkine correlated with increased myeloid diversity and its plasma concentration was predictive of the benefit from ICI in NSCLC [36]. Further studies are warranted to confirm the hypothesis that high CX3CL1 expression contributes to increased benefit from ICI in lung adenocarcinoma, to reveal the origin of CX3CL1 that could either be expressed by the tumour cells or by cell of the TME and to decipher the connection of tumour genetics and CX3CL1 expression.

The study has the following limitations: First, the study was limited to KRAS and TP53 as the two most frequently mutated genes in lung adenocarcinomas. A recent systematic study based on WES identified ATM mutations as a positive predictive marker for response to ICI in NSCLC [15]. A larger number of negative predictive markers were suggested including EGFR mutations and ALK fusions [37], STK11 mutations [11, 38, 39], as well as KEAP1 and NFE2L2 mutations [40]. STK11 and KEAP1 mutations were associated with worse outcomes to immunotherapy in KRASmut but not in KRASwt lung adenocarcinoma [41]. Multiple gene models including interaction terms between pairs of genes outperformed models without interaction terms in the prediction of immunotherapy efficiency [42]. An optimised panel of genes interrogating mutations and mutation combinations that serve as positive and negative predictors of ICI benefit still remains to be defined.

Second, because of the retrospective nature of the study without a control arm, it was not possible to distinguish between predictive and prognostic markers. To overcome this limitation, we analysed two cohorts of surgically treated lung adenocarcinoma patients who did not receive ICI as early treatment. However, these cohorts included predominantly stage I-III tumours compared with the three ICI cohorts that included exclusively stage IV tumours. It was a strength of the current study to have the opportunity to study a large real-world cohort with a total of more than 700 patients and more than 120 patients with KRAS/TP53 double-mutated tumours from a single institution.

Today, several approved first-line PD-(L)-1 blockade regimens alone or in combination with platinum-based chemotherapy are available for advanced lung adenocarcinoma, but clinical trials comparing these regimens have not been conducted. In a few retrospective studies, the population of never-smokers with PD-L1-high ( ≥ 50%) expression had better PFS and OS outcomes when chemotherapy was added [43–45]. Additional biomarkers for treatment guidance would be of value. Based on the predictivity demonstrated here and elsewhere [12, 13], dual KRAS/TP53 mutation should be further investigated, in particular as a biomarker for sparing chemotherapy.

Comprehensive analysis of multiple datasets identified dual KRAS/TP53 mutation as a predictive biomarker for ICI, which is associated with unique tumour molecular characteristics and can be easily interrogated by small gene panels or even single gene analysis. Future studies are warranted to substantiate these findings and evaluate the potential of this biomarker for therapy guidance.

### Supplementary information

Supplemental Material

## Data Availability

The data generated in this study are available upon request from the authors.

## Code availability

The R code for statistical analysis and generation of the figures is available upon request from the authors.
