# Supplementary material for: KRAS and TP53 co-mutation predicts benefit of immune checkpoint blockade in lung adenocarcinoma
Source: Br J Cancer. 2024 Jun 12;131(3):524–33. doi: 10.1038/s41416-024-02746-z (PMC11300455; doi:10.1038/s41416-024-02746-z)
Supplement: Supplementary file 1 — Supplemental Material [file 41416_2024_2746_MOESM1_ESM.pdf]

Supplemental Material: KRAS and TP53 co-mutation predicts benefit of immune checkpoint blockade in lung adenocarcinoma

Supplemental Data 1: Clinicopathological characteristics of the Heidelberg cohort of lung adenocarcinoma patients treated with immunotherapy (HD-ICI) and of the TCGA cohort of conventionally treated lung adenocarcinoma patients (TCGA-LUAD). Tumors harboring EGFR driver mutations or ALK/RET/ROS1 driver fusions were excluded from the cohorts.

Variable	Value	HD-ICI	TCGA-LUAD
**Number of patients**		713	417
**Age**		65 ± 8.9	65.3 ± 10.3
**Sex**			
	male	396 (56%)	205 (49%)
	female	317 (44%)	212 (51%)
**Smoking**			
	smoker	603 (85%)	366 (88%)
	non-smoker	110 (15%)	39 (9.4%)
	NA	0 (0%)	12 (2.9%)
**Stage**			
	I	0 (0%)	229 (55%)
	II	0 (0%)	99 (24%)
	III	0 (0%)	64 (15%)
	IV	713 (100%)	20 (4.8%)
	NA	0 (0%)	5 (1.2%)
**PD-L1**			
	≥ 50%	206 (29%)	N/A
	≥ 1-49%	279 (39%)	N/A
	negative	203 (28%)	N/A
	NA	25 (3.5%)	N/A
**Therapy**			
	ICI monotherapy	314 (44%)	0 (0%)
	ICI and CHT	399 (56%)	0 (0%)
	w/o ICI	0 (0%)	417 (100%)
**Therapy line ICI**			
	1st line	465 (65%)	N/A
	≥ 2nd line	248 (35%)	N/A

Supplemental Data 2: Overview of the analyzed cohorts of lung adenocarcinoma patients.

Cohort	n	Treatment	PubMed ID	Reference
**HD-ICI**	713	ICI	--	Thoraxklinik Heidelberg
**SU2C-ICI**	247	ICI	37024582	Ravi et al., Nat Genet. 2023 May, 55(5)
**MSK-ICI**	135	ICI	36038778	Vanguri et al., Nat Cancer. 2022, 3(10)
**TCGA-LUAD**	417	surgery	25079552	Cancer Genome Atlas Research Network, Nature, 2014, 511(7511)
**MSK-LUAD**	394	surgery	32791233	Caso et al., J Thorac Oncol. 2020, 15(12)

Supplemental Data 3: Inclusion and exclusion of patients in the analyzed cohorts HD-ICI, SU2C-ICI, MSK-ICI, TCGA-LUAD, and MSK-LUAD.

Inclusion / exclusion	HD-ICI	SU2C-ICI	MSK-ICI	TCGA-LUAD	MSK-LUAD
**Total number of adenocarcinomas**	**875**	**286**	**183**	**515**	**604**
**Excluded: tumors with EGFR mutation**	0	14	15	68	182
**Excluded: tumors with ALK/RET/ROS1 fusion**	0	0	1	11	20
**Total: EGFR and ALK/RET/ROS negative adenocarcinomas**	875	272	167	436	402
**Excluded: patients that progressed or died within six weeks**	162	25	32	19	8
**Total: patients included in survival analyses**	**713**	**247**	**135**	**417**	**394**

Supplemental Data 4: Association of KRAS and TP53 co-mutation status with patients and tumor characteristics in the TCGA cohort of 417 stage I-IV lung adenocarcinoma (TCGA-LUAD).

Variable	Value	KRASwt/ TP53wt	KRASwt/ TP53mut	KRASmut/ TP53wt	KRASmut/ TP53mut	p
**Prevalence**		116 (27.8%)	158 (37.9%)	93 (22.3%)	50 (12%)	
**Age**	mean ± sd	68 ± 9.2	63.7 ± 10.5	65.8 ± 10.4	63.4 ± 10.5	0.0027
**Sex**						0.054
	female	65 (30.7%)	70 (33%)	45 (21.2%)	32 (15.1%)	
	male	51 (24.9%)	88 (42.9%)	48 (23.4%)	18 (8.8%)	
**Smoking**						0.0013
	smoker	91 (24.9%)	143 (39.1%)	85 (23.2%)	47 (12.8%)	
	non-smoker	22 (56.4%)	10 (25.6%)	5 (12.8%)	2 (5.1%)	
**Tumor stage**						0.61
	Stage I	74 (32.3%)	85 (37.1%)	46 (20.1%)	24 (10.5%)	
	Stage II	21 (21.2%)	39 (39.4%)	25 (25.3%)	14 (14.1%)	
	Stage III	16 (25%)	24 (37.5%)	16 (25%)	8 (12.5%)	
	Stage IV	4 (20%)	8 (40%)	4 (20%)	4 (20%)	

Supplemental Data 5: Influence of KRAS and TP53 co-mutation status on PFS in patients treated with ICI. Univariate and multivariate analyses in HD-ICI.

Variable	Value	HR PFS univariate	p PFS univariate	HR PFS multivariate	p PFS multivariate
**Tumor genetics**					
	KRASmut	0.91 (0.73-1.13)	0.39	1.01 (0.8-1.27)	0.93
	TP53mut	1.07 (0.84-1.35)	0.59	1.2 (0.93-1.54)	0.16
	interaction	0.72 (0.51-1.02)	0.068	0.69 (0.48-0.99)	0.045
**Age**					
	per 10 years	1.05 (0.96-1.16)	0.28	1.06 (0.96-1.17)	0.25
**Sex**					
	female vs. male	0.91 (0.77-1.08)	0.27	1.13 (0.94-1.35)	0.19
**Smoking**					
	smoker vs. never/light	0.78 (0.62-0.98)	0.032	0.83 (0.65-1.07)	0.15
**PD-L1**					
	≥ 50% vs. negative	0.54 (0.43-0.68)	9.7e-08	0.5 (0.38-0.64)	1.4e-07
	≥ 1-49% vs. negative	0.76 (0.62-0.93)	0.0083	0.74 (0.6-0.92)	0.0058
**Comb. with CHT**					
	ICI + CHT vs. ICI	0.97 (0.81-1.15)	0.69	1.14 (0.89-1.45)	0.3
**Therapy line ICI**					
	1st line vs. ≥ 2nd line	0.79 (0.66 -0.94)	0.0072	0.81 (0.64-1.03)	0.082

Supplemental Data 6: Influence of KRAS and TP53 co-mutation status on OS in patients treated with ICI. Univariate and multivariate analyses in the HD-ICI cohort.

**Variable**	**Value**	**HR OS univariate**	**p OS univariate**	**HR OS multivariate**	**p OS multivariate**
Tumor genetics					
	KRASwt/TP53wt	1.38 (1.03-1.84)	0.029	1.06 (0.77-1.47)	0.72
	KRASwt/TP53mut	1.65 (1.21-2.24)	0.0014	1.57 (1.14-2.16)	0.0054
	KRASmut/TP53wt	1.28 (0.95-1.74)	0.11	1.28 (0.95-1.73)	0.1
Age					
	per 10 years	1.16 (1.04-1.29)	0.0073	1.17 (1.05-1.31)	0.0063
Sex					
	female vs. male	0.86 (0.71-1.04)	0.13	1.13 (0.92-1.38)	0.24
Smoking					
	smoker vs. never/light	0.83 (0.64-1.06)	0.14	0.89 (0.67-1.18)	0.42
PD-L1					
	≥ 50% vs. negative	0.61 (0.47-0.79)	0.00014	0.58 (0.44-0.78)	0.00024
	≥ 1-49% vs. negative	0.82 (0.65-1.03)	0.091	0.8 (0.63-1.02)	0.068
Combination with CHT					
	ICI + CHT vs. ICI	1 (0.83-1.21)	0.98	1 (0.76-1.31)	0.98
Line of therapy					
	1st vs. ≥ 2nd line	0.85 (0.7-1.03)	0.091	0.81 (0.63-1.05)	0.11

Supplemental Data 7: Influence of KRAS and TP53 co-mutation status on PFS in patients treated with ICI. Univariate and multivariate analyses in the HD-ICI cohort.

**Variable**	**Value**	**HR PFS univariate**	**p PFS univariate**	**HR PFS multivariate**	**p PFS multivariate**
Tumor genetics					
	KRASwt/TP53wt	1.3 (1-1.68)	0.046	1.2 (0.9-1.59)	0.21
	KRASwt/TP53mut	1.52 (1.16-2.01)	0.0027	1.43 (1.08-1.91)	0.013
	KRASmut/TP53wt	1.43 (1.09-1.86)	0.0087	1.21 (0.93-1.58)	0.16
Age					
	per 10 years	1.05 (0.96-1.16)	0.28	1.06 (0.96-1.17)	0.25
Sex					
	female vs. male	0.91 (0.77-1.08)	0.27	1.13 (0.94-1.35)	0.19
Smoking					
	smoker vs. never/light	0.78 (0.62-0.98)	0.032	0.83 (0.65-1.07)	0.15
PD-L1					
	≥ 50% vs. negative	0.54 (0.43-0.68)	9.7e-08	0.5 (0.38-0.64)	1.4e-07
	≥ 1-49% vs. negative	0.76 (0.62-0.93)	0.0083	0.74 (0.6-0.92)	0.0058
Combination with CHT					
	ICI + CHT vs. ICI	0.97 (0.81-1.15)	0.69	1.14 (0.89-1.45)	0.3
Line of therapy					
	1st vs. ≥ 2nd line	0.79 (0.66 -0.94)	0.0072	0.81 (0.64-1.03)	0.082

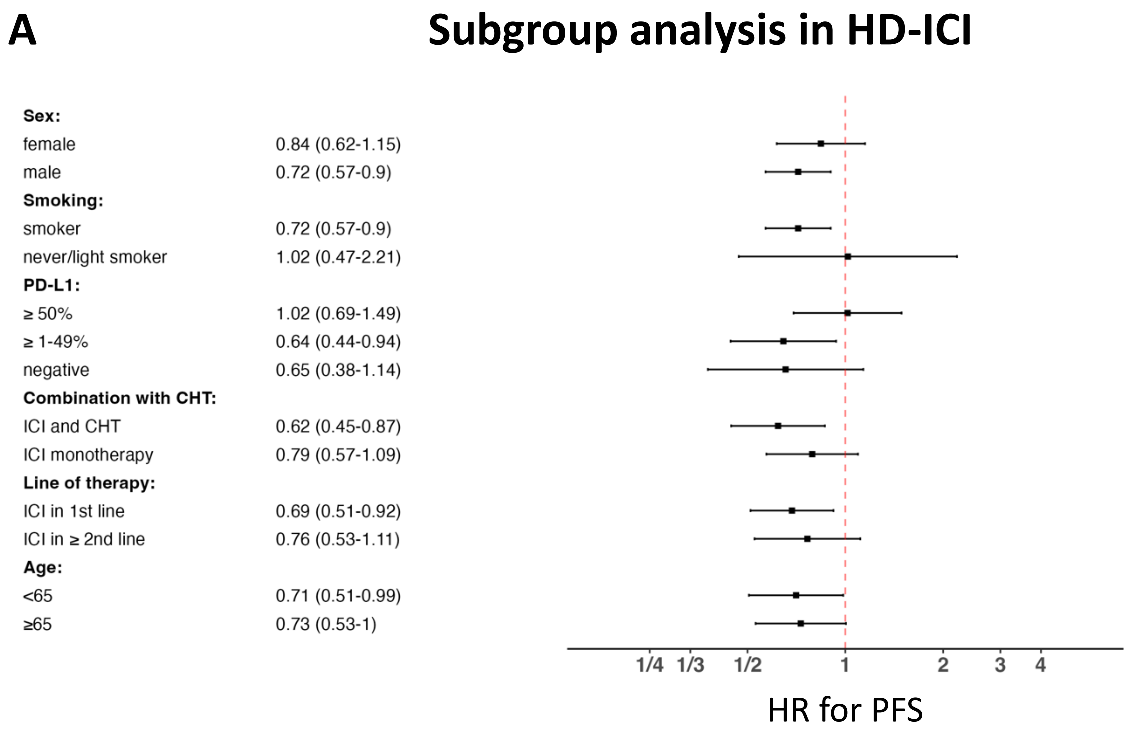

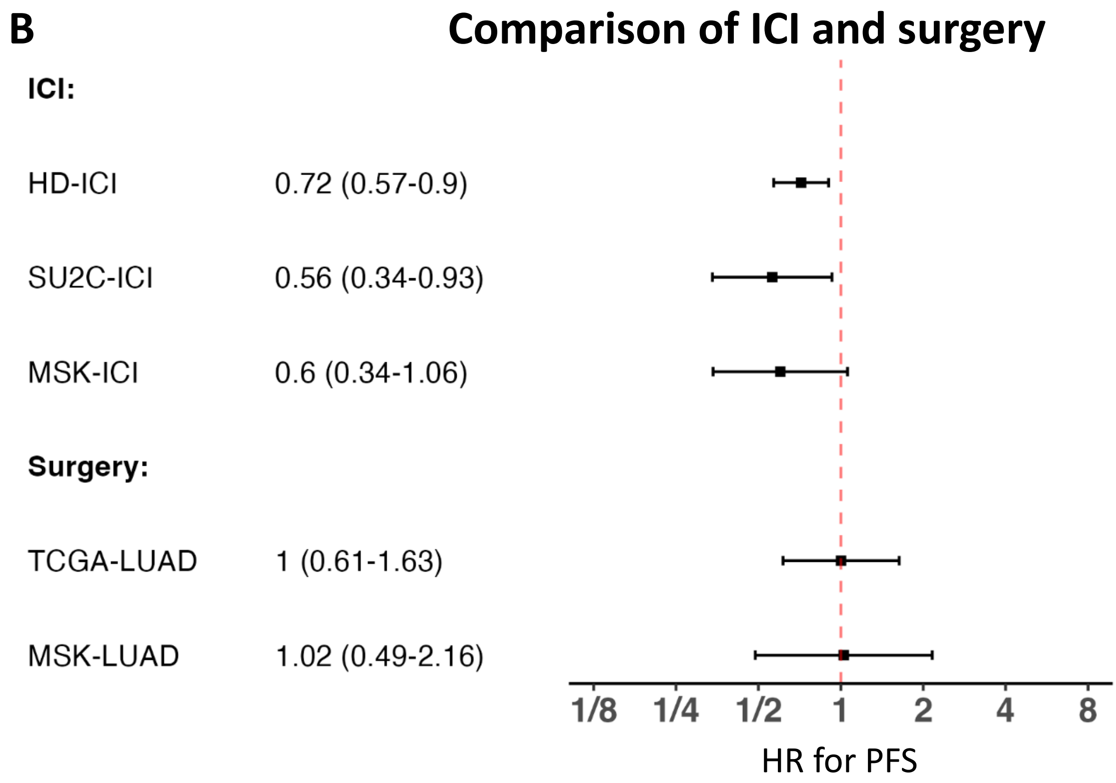

Supplemental Data 8: KRAS and TP53 co-mutation as predictive marker for benefit from ICI. PFS of double-mutated tumors in comparison to tumors not harboring both mutations. **A** Subgroup analysis in the Heidelberg cohort of patients treated with ICI (HD-ICI). **B** Comparison of ICI and conventional therapy. Cohorts of stage IV lung adenocarcinoma patients treated with ICI: HD-ICI, SU2C-ICI, and MSK-ICI. Cohorts of conventional treated lung adenocarcinoma patients: TCGA-LUAD and MSK-LUAD.

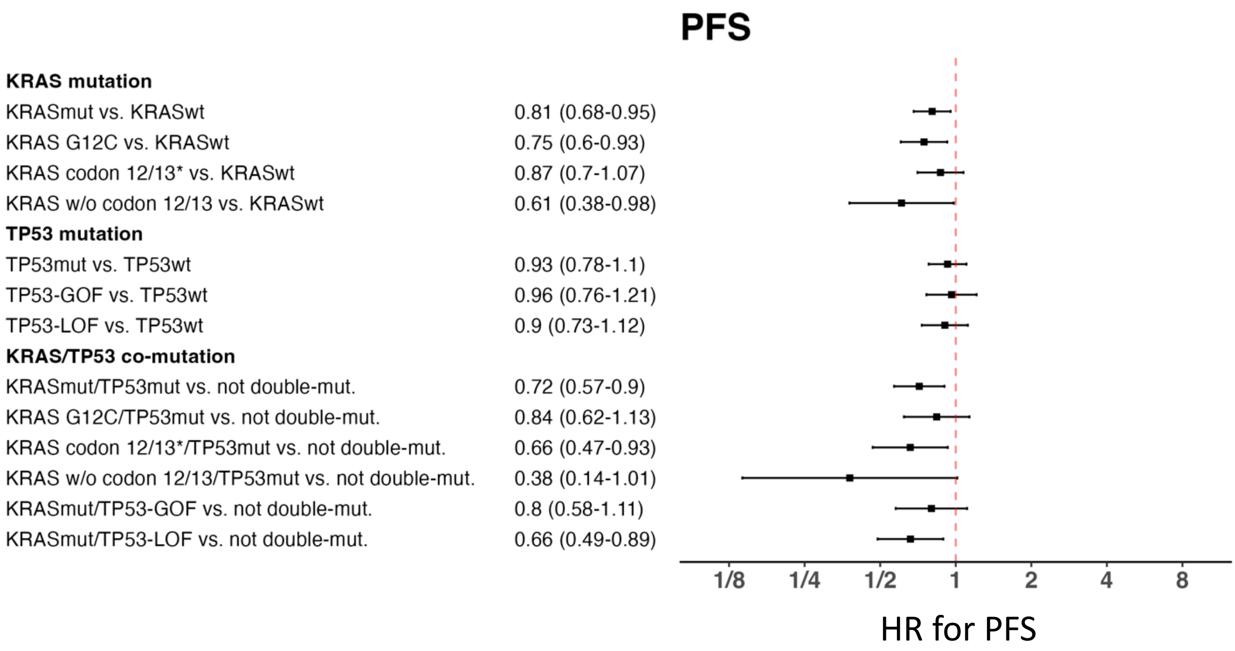

Supplemental Data 9: Univariate analysis of PFS comparing tumors having specific KRAS mutations with KRASwt tumors, of tumor having specific TP53 mutations with TP53wt tumors, and of tumors having specific types of KRAS/TP53 co-mutation with the basket of not double-mutated tumors. * = without G12C mutation

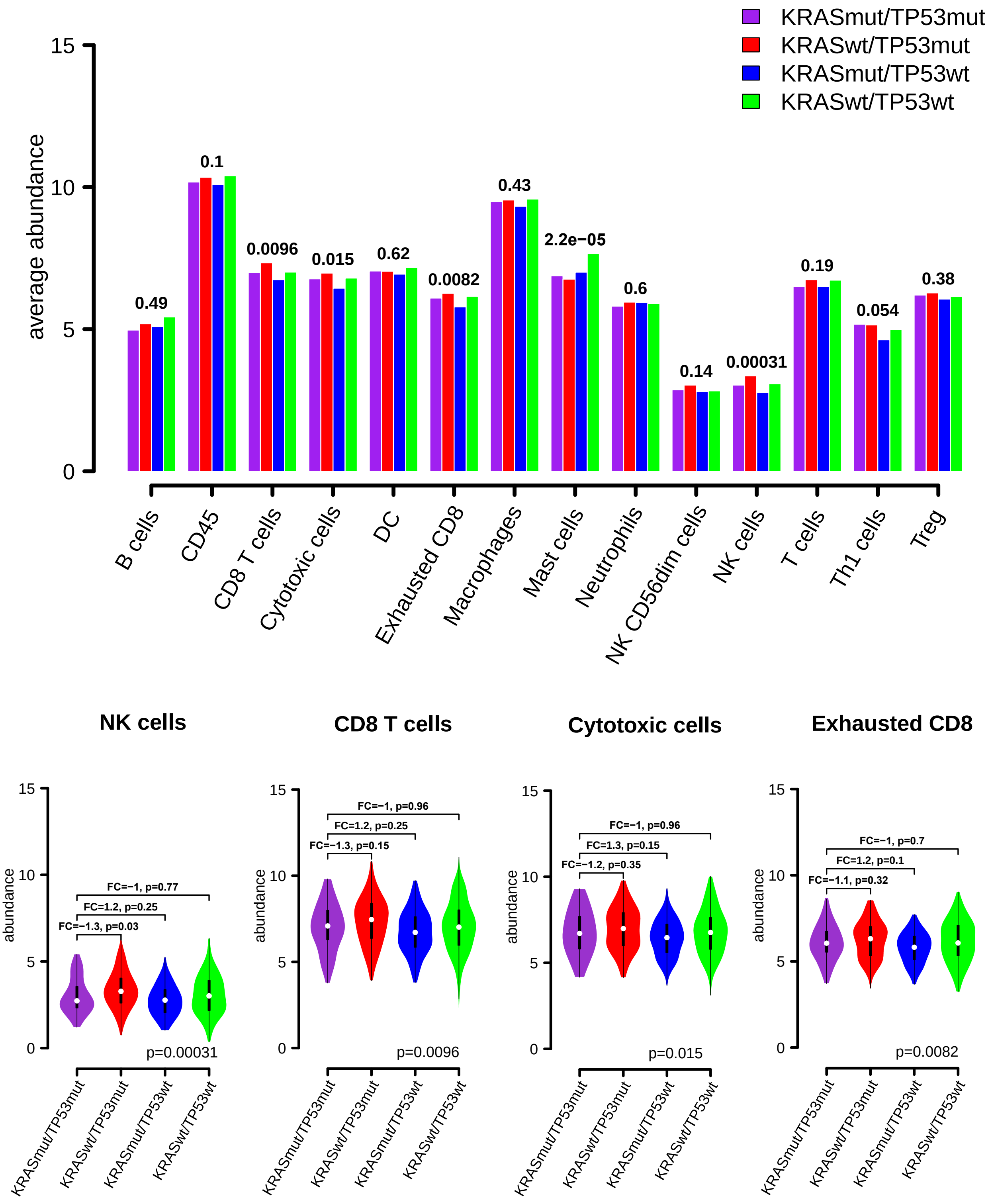

Supplemental Data 10: Analysis of the association of the abundance 14 immune cell population with KRAS and TP53 co-mutations status in TCGA-LUAD. Number above the grouped bars refer to p-values assessing the difference between the four mutation configurations.

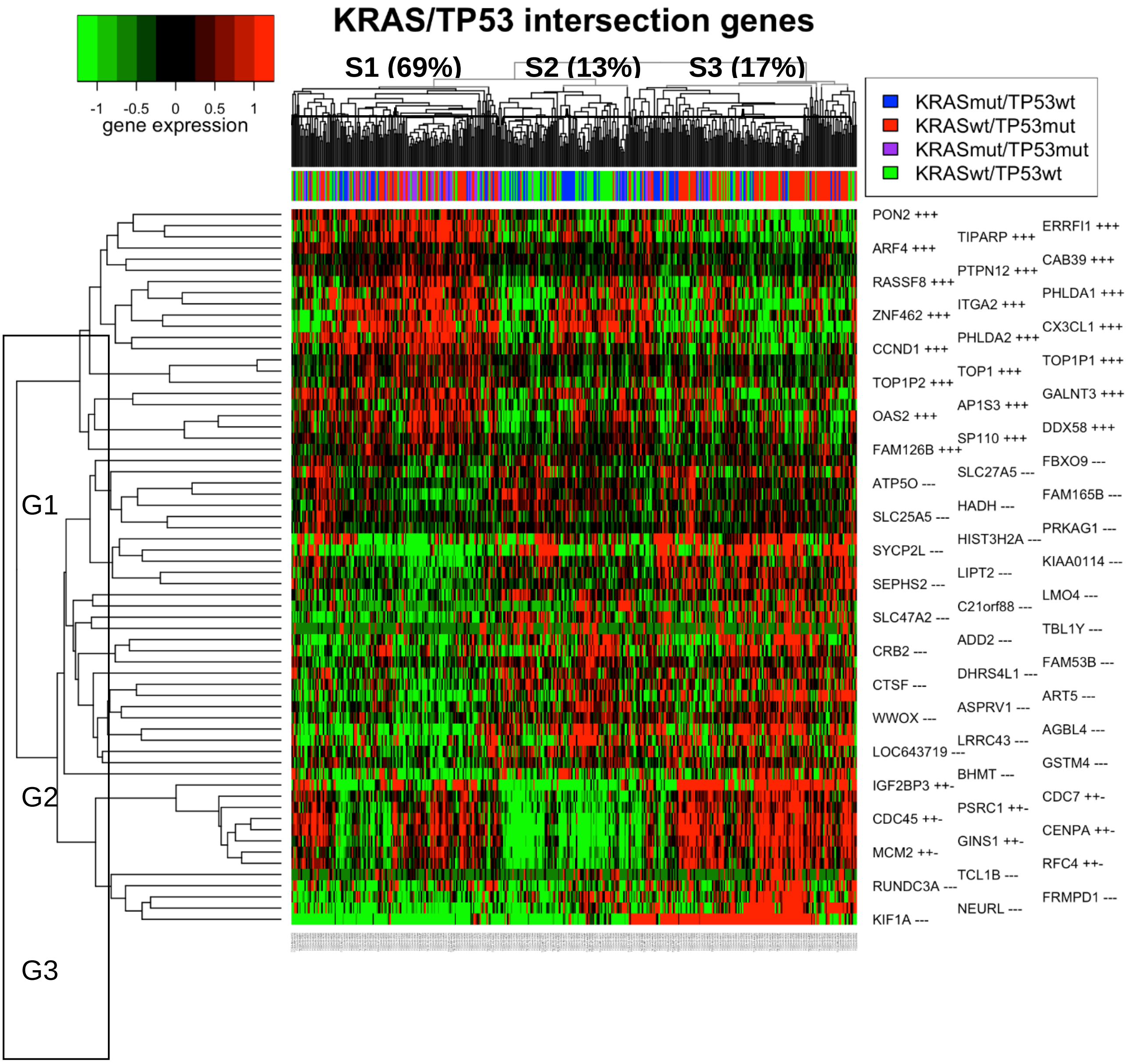

Supplemental Data 11: Heatmap of the 64 genes that distinguished double-mutation from the other three mutation configurations. Most (69%) of the double-mutated tumors exhibited the expression pattern of cluster C1. Signs behind the genes refer to overexpression (+) or underexpression (-) in the comparison of double mutated tumors with KRASmut/TP53wt, KRASwt/TP53wt, and KRASwt/TP53mut tumors.

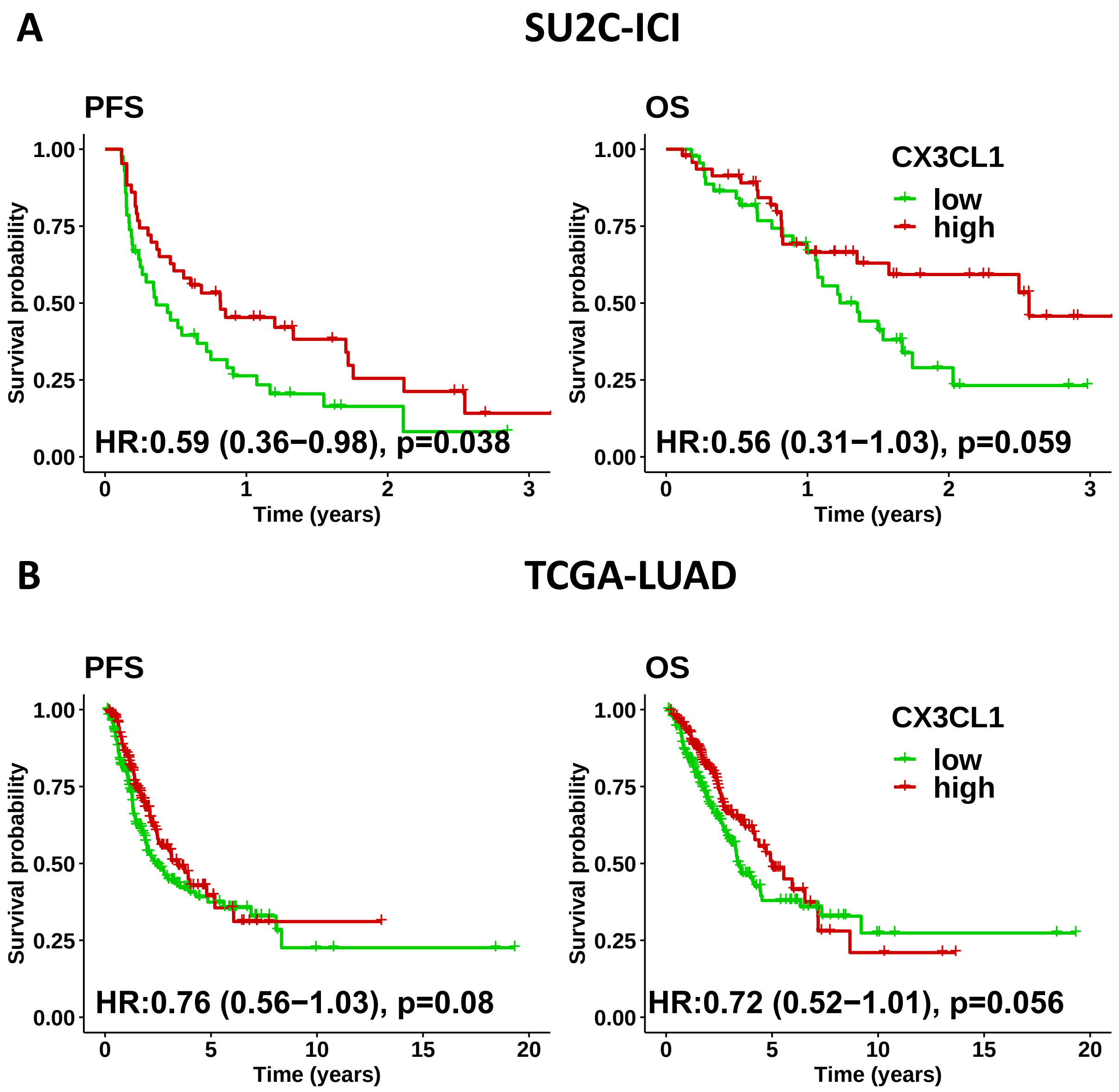

Supplemental Data 12: CX3CL1 mRNA expression as prognostic marker in lung adenocarcinoma. Patients were stratified into high and low expression using the median expression as cutpoint. **A** Analysis of patients treated with immunotherapy (SU2C-ICI). **B** Analysis of surgical treated patients (TCGA-LUAD).
